# Efficiency of a Modified Ovulation Synchronization Program in the Treatment of Ovarian Cysts in Dairy Cattle

**DOI:** 10.3390/ani15070995

**Published:** 2025-03-30

**Authors:** Daniela Haldi, Eveline Studer, Esther Rothenanger, Jürg Hüsler, Adrian Steiner, Gaby Hirsbrunner

**Affiliations:** 1 Clinic for Ruminants, Vetsuisse Faculty, University of Bern, Bremgartenstrasse 109a, CH-3012 Bern, Switzerland; daniela.haldi@unibe.ch (D.H.); eveline.studer@unibe.ch (E.S.); adrian.steiner@unibe.ch (A.S.); 2 LaboRvet, Wiggermatte 16, CH-6260 Reiden, Switzerland; esther.rothenanger@laborvet.ch; 3 Institute of Mathematical Statistics and Actuarial Science, University of Bern, Sidlerstrasse 5, CH-3012 Bern, Switzerland; juerg.huesler@stat.unibe.ch

**Keywords:** cattle, ovarian cyst, therapy, modified ovsynch protocol

## Abstract

In dairy cattle, ovarian cysts (COFs) represent a major cause of infertility. They can be divided morphologically into follicular and luteal cysts based on their wall thickness, which can be examined by ultrasound, and progesterone secretion, which can be analyzed in serum or milk. The aim of our study was to evaluate cyst recovery using a modified ovsynch protocol with no need to differentiate COFs. Additionally, the beta-hydroxybutyric acid level (BHB), progesterone values, and trace elements in the serum were measured when therapy started. Fourteen days after treatment, COF recovery was confirmed in 88% of the cases. The median calving-to-conception interval, number of artificial inseminations until pregnancy, and median number of days from treatment to pregnancy were not different between the modified ovsynch protocol group and all other COF treatments. The logistic regression for COF included the parameters group, the cyst type, breed, the number of artificial inseminations (AIs), calving to conception cut at 200 d p.p., the cyst size, and therapy. The backward (and also forward) variable selection of the logistic regression yielded only the cyst size as a significant negative impact factor for recovery. In conclusion, the modified ovsynch protocol is a useful, practical option for COF treatment with the advantage of not needing to differentiate between the two cyst types.

## Simple Summary

In dairy cattle, ovarian cysts represent a serious dysfunction of the ovaries. Thin-walled follicular cysts and luteinized cysts exhibiting thicker walls are described. The diagnosis is based on examination by ultrasound and serum or milk progesterone analysis. For a practitioner, therapies without differentiating cysts would be warranted. Therefore, the effectiveness of a modified ovsynch protocol in which differentiation was not needed was evaluated. Fourteen days after treatment, cyst recovery was confirmed in >88% of the cows treated. The median calving-to-conception interval was 95 d. A logistic regression analysis revealed that only larger cyst sizes negatively influenced cyst regression. The modified ovsynch protocol is a useful, practical option for treating ovarian cysts with the advantage of not needing to differentiate between the two cyst types. It can be applied under field conditions and allows for the possibility of timed artificial insemination.

## 1. Introduction

In dairy cattle, ovarian cysts are the most common dysfunctions of the ovaries and represent a major cause of infertility [1,2,3]. One important effect on reproductive performance is the extension of the intercalving interval, with a prolongation of the calving-to-conception interval [4,5]. Treatment costs and a higher culling rate increase economic losses [1,5,6]. The incidence of ovarian cysts varies from 2.7 to 30% across studies [3,7,8,9,10,11]. Most of the cysts observed occur between 30 and 60 d postpartum (p.p.), with another increase in incidence after 120 d p.p. [7,8,9,10].

During the ovarian cycle in cattle, two or three follicular waves occur [11]. From each wave, one dominant follicle is selected, which continues to grow, while the other follicles regress [12]. A typical characteristic of the dominant follicle is its ability to secrete estradiol leading to decreased Follicle-Stimulating Hormone (FSH) levels below the minimum demand of the smaller follicles, leading to their regression [13]. Cystic ovarian follicles (COFs) arise from one or more follicles that neither ovulate nor regress but continue to grow and produce steroids [14]. A study has shown that COFs secrete estradiol for an average of 17 d but are detectable by ultrasonography for up to 52 d [15]. COFs are dynamic structures that can persist on the ovaries for a longer period; they can regress and be replaced by new COFs, or they can allow for the development of a new ovarian cycle through spontaneous healing [5].

The exact pathogenesis of COFs is not yet fully understood. Dysfunction of the hypothalamic–pituitary–ovarian axis seems to lead to cyst formation. An altered luteinizing hormone (LH) released from the hypothalamus–pituitary is the most widely acknowledged hypothesis, meaning that the peak of LH, which normally provokes ovulation, is either completely absent, insufficient, or arises at the wrong time. This finally leads to ovulation failure and, hence, the formation of a COF [14].

In rectal ultrasound examinations, COFs can be morphologically subdivided into follicular and luteal cysts, with follicular cysts being more common [16]. Follicular cysts are thin-walled (≤3 mm) with anechogenic follicular fluid, whereas luteal cysts have a thicker luteal wall (>3 mm) and often show hyperechogenic structures within the follicular fluid [14]. Luteal cysts are believed to be follicular cysts in later stages, where, on a cellular level, thecal and granulosa cells from a follicular cyst have luteinized and secrete progesterone [8], whereas follicular cysts secrete little to no progesterone [8,17]. The correct functional diagnosis of the cyst type requires progesterone assessment in addition to ovarian examination by ultrasound [18].

In the past, various authors defined follicular cysts as structures with a diameter of at least 25 mm, persisting on the ovaries for a minimum of 10 d [15,19]. Different studies showed that dominant follicles may ovulate at a much smaller size than 25 mm (14.3 to 18.6 mm) [20,21]. Silvia et al. (2002) defined ovarian cysts with a diameter of at least 17 mm and persisting for more than 6 d [1]. In the present study, we used the definition of Vanholder et al. (2006), defining follicular cysts as follicles with a diameter of at least 20 mm that are present on one or both ovaries in the absence of active luteal tissue and interfering with normal ovarian cyclicity [14].

Risk factors associated with the increased occurrence of COFs are high milk yield, whereby the probability for cyst formation increases with higher milk production; calving season; an abnormal puerperal phase (e.g., twins, retained placenta, primary metritis, and ketonuria), gain in body condition status (BCS) prepartum, and lactation number [22,23].

Between 38 and 60% of COFs that develop during the early p.p. period regress spontaneously without medical treatment [10,23]. For a practitioner, it is challenging to decide when and if to treat COFs instead of anticipating spontaneous recovery.

The treatment of COFs in cows aims to restore or induce an ovulatory surge of LH, which will lead to the ovulation or luteinization of a functional follicle [24]. Therapy can be implemented using substitution therapy with gonadotropin-releasing hormone (GnRH) or human chorionic gonadotropin (hCG) [5,25,26]. The use of GnRH leads to estrus about 3 weeks after initiating the treatment [27,28] with a recovery rate of 70 to 75% [29,30]. Another treatment option is the use of an ovulation synchronization (ovsynch) protocol with GnRH and Prostaglandin F_2α_ (PGF_2α_) [25]. However, pregnancy rates for cystic cows undergoing treatment with an ovsynch protocol are low, ranging from 21 to 27% [25,29,31]. Modified ovsynch protocols using a simultaneous administration of GnRH and PGF_2α_ seem to improve treatment success compared to GnRH alone [32]. A possible synergistic effect of PGF_2α_ and GnRH in the luteinization of follicular cysts and a better recovery rate have been shown [32]. In contrast, Taktaz et al. (2015) could not demonstrate the above-mentioned advantage and reported no improvement in reproductive performance after using GnRH or hCG and PGF_2α_ simultaneously; however, 36.1% of those cows were treated earlier than 40 d p.p. [28]. The administration of progesterone via an intravaginal device is an effective way to treat both follicular and luteal cysts, and in one study led to recovery in 82% (n = 14 cows with follicular cysts) and 70% (n = 7 cows with luteal cysts) and conception rates of 53.8%/71.4% [33]. Luteal cysts generally respond to PGF_2α_ due to its luteolytic effect [5]. The administration of PGF_2α_ will lead to estrus within 8 d in 87 to 96% of treated patients [5].

Part A of the current study focuses on the effectiveness of a treatment regimen for COFs in dairy cows in practice, using a modified ovsynch protocol. Clinical cure, the calving-to-conception interval, and the number of artificial inseminations (AIs) to pregnancy were evaluated and also compared to routine cyst treatments used on the same farms during the same time period.

Part B of the current study evaluated risk factors (e.g., the size of the cyst, the beta-hydroxybutyric acid level (BHB), trace element levels, and BCS-loss) potentially contributing to the development of cysts in the group where the modified ovsynch protocol was used.

## 2. Materials and Methods

### 2.1. Farms

The study was performed on 31 dairy farms in Switzerland, which were visited every two weeks for routine reproduction, udder health, and nutrition management by the Herd Health Service of the Clinic for Ruminants, Vetsuisse Faculty, University of Bern. The farms had dairy cows of various breeds, mainly Holstein Friesian, Red Holstein, and Swiss Fleckvieh, as well as a few Jersey, Braunvieh, Montbéliard, and Grauvieh cows. The number of lactating cows varied between 12 and 77 animals per herd. The cows were kept in tie and free stalls. The average herd milk yield (standard lactation of 305 d) was between 6100 kg and 9900 kg. Production data including the data of individual animals were collected from the herd health software used by the Herd Health Service (DSA Laitier vétérinaire 32, version 11.12, Saint Hyacinthe, QC, Canada). Farms performing seasonal calving were excluded from the study.

### 2.2. Animals, Definitions and Including Criteria

The study was conducted during a period of 14 months (1 August 2021 to 30 September 2022). The cows (n = 1067) underwent a routine p.p. examination, both vaginally by hand or using a speculum and transrectally, including ultrasonography (linear probe, 7.5 MHz, Draminski, iScan or iScan2, Szabruk, Poland), 21 to 35 d after calving, or a later gynecological exam was conducted after 35 d p.p. to determine their current state of cyclicity. COFs were defined as follicular structures with a diameter of at least 20 mm in the absence of a corpus luteum [14]. The cows in the present study were examined every 14 d as part of the routine work of the Herd Health Service team. Cows were included if cysts were diagnosed between d 21 and 100 of lactation. Animals that had a history of cesarean section, uterine torsion, uterine prolapse, or displacement of the abomasum (within the first 5 weeks after calving) were excluded. Cows more than 100 d p.p. when first diagnosed with COFs were also excluded.

### 2.3. Study Design and Treatment

**Figure 1:**
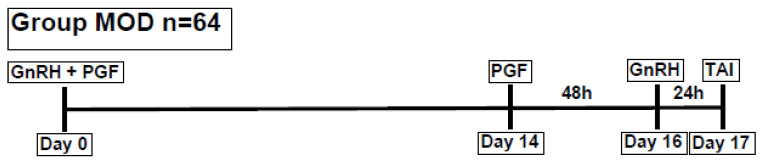
Modified ovsynch protocol (GnRH = gonadotropin-releasing hormone, PGF = prostaglandin F_2α_, TAI = timed artificial insemination, and n = number of animals).

If COFs were diagnosed, the diameter and wall thickness of the cysts were measured by ultrasonography, a blood sample was taken to measure BHB levels, and the body condition score (BCS) was determined. Cows diagnosed with COFs were re-examined 14 d later to confirm the persistency of the cyst/anovulatory condition. If this was the case, they were treated using a modified ovsynch protocol as described by Gundling et al. 2015 (group MOD) [31]. On day 0, the cows received 0.02 mg of buserelin i.m. (Buserol^®^, Dr. E. Graeub AG, Bern, Switzerland) and 0.15 mg of cloprostenol i.m. (Genestran^®^, Dr. E. Graeub AG, Bern, Switzerland), followed by 0.15 mg of cloprostenol i.m. on day 14 and 0.02 mg of buserelin i.m. on day 16. Timed artificial insemination (TAI) was performed 20 to 24 h after the second buserelin injection (Figure 1).

Not all cows were treated according to the modified ovsynch protocol during the study period. There were various reasons why another therapy was used (e.g., GnRH alone, hCG alone, or combined protocols with an intravaginal progesterone device (EAZI-BREED CIDR 1380^®^, Zoetis Schweiz GmbH, Delémont, Switzerland)). That group of animals (n = 81) served as the routinely treated group (ROUT).

The earliest start of the modified ovsynch protocol was on day 42 after calving, giving the first possible date for a TAI on day 59 after calving. The calving-to-conception interval was cut at 200 d p.p., meaning animals more than 200 d p.p. were excluded from the study. We assumed that the occurrence of COFs in a later stage of lactation has other risk factors and causes than the ones appearing in the first months of lactation. Other studies concerning fertility problems in cows have also excluded cows more than 200 d p.p. or allocated them to a separate group [34,35].

### 2.4. Laboratory Variables (Part B of the Study)

For each COF diagnosis during the study period, a blood sample was taken from the tail vein to measure the BHB level using a rapid cowside test (BHB check, Streuli Tiergesundheit AG, Uznach, Switzerland). BHB was categorized into normal (<1.2 mmol/L), subclinical ketosis (1.2–2.9 mmol/L), and clinical ketosis (>2.9 mmol/L) [36]. In the cows of group MOD, three blood samples were taken: two samples were collected immediately before the 1st treatment. In addition to the BHB analysis, one sample was used to measure progesterone levels (LH 170 I.U. BD Vacutainer^®^, Becton Dickinson, CH-4123 Allschwil, Switzerland), and the second sample was collected in a K2 EDTA 10.8 mg BD Vacutainer^®^, Becton Dickinson, CH-4123 Allschwil, Switzerland to analyze trace elements (iodine, manganese, zinc, selenium, and copper). On day 14, a third blood sample was collected to measure the BHB, as well as the progesterone level. The blood samples were centrifuged, and the serum was frozen at −20 °C until the end of the study. Then, all samples were sent to a laboratory (laboRvet AG, Reiden, Switzerland) for an analysis of progesterone and trace elements. Analysis of progesterone was performed using a chemiluminescence immunoassay (Siemens Immulite 2000XPi, Siemens, CH-8047-Zürich, Switzerland); manganese was measured using atomic absorption, whereas the other trace elements (iodine, zinc, selenium, and copper) were measured using ICP-MS mass spectrometry. Laboratory analyses were only performed for the group MOD. Additionally, the BCS was determined during the examination 21–35 d p.p. and 14 d later. It was scored visually using a system ranging from 1 (=severely underconditioned) to 5 (=severely over-conditioned) [37].

The threshold for progesterone in serum was 1 ng/mL for luteal activity [38]. Thresholds for trace elements were the following: iodine in umol/l (>48, normal; 32–48, lower normal range; ≤31, deficiency); selenium in umol/l (>49, normal; 30–49, lower normal range; ≤29, deficiency); zinc in umol/L (>850, normal; 654–850, lower normal range; ≤653, deficiency); copper in umol/L (>766, normal; 391–766, lower normal range; ≤390, deficiency); manganese in nmol/l (>1.7, normal; 1.3–1.7, lower normal range; ≤1.2, deficiency) [39].

### 2.5. Follow-Up and Outcome

After the completion of the cyst treatment in both groups, the cows were examined gynecologically to evaluate the efficacy of the treatment. If cows were inseminated, a transrectal ultrasound was performed starting 28 d after insemination to check for a possible pregnancy.

As a short-term outcome, recovery 14 d after the initial COF treatment was evaluated. Recovery was defined as either estrus, estrus and artificial insemination (AI) within 14 d after treatment, or no COF identified 14 d after treatment. The following variables were evaluated as a long-term outcome: the calving-to-conception interval, the interval from day 0 of cyst treatment to the first day of pregnancy, and the number of AIs per pregnancy.

### 2.6. Statistical Analysis

For categorical data, the frequency of categories was determined. In metric variables, mean, median, sd, 25% and 75% quartiles, minimum, and maximum were calculated. Frequencies in categorical data were assessed using the Chi-square test. Nonparametric tests were used (Wilcoxon–Mann–Whitney test) for the analysis of metric data. A *p*-value < 0.05 indicated a significant result. The *p*-value was not corrected for multiple testing. Data were analyzed using the statistical software SAS^®^ version 9.4 (SAS Institute Inc., Cary, NC, USA, Available online: www.sas.com). Multiple logistic regression analysis with backward elimination was conducted to determine risk factors that affected the occurrence of COFs.

## 3. Results

A total of 64 cows were treated with the modified ovsynch protocol, and 81 animals received another treatment for COFs out of a total of 1067 cows undergoing a p.p. examination (Table 1). Because of some missing data, the sample size was not always 64 and 81. The ROUT group consisted of GnRH or hCG (n = 51 cows), PGF2a (n = 9 cows), and combined protocols together with EAZI-BREED CIDR 1380^®^ (21 cows). During the same period, another 131 cows were diagnosed with COFs but were left untreated (too early p.p., not expected to stay productive, and extended voluntary waiting period). These cows were not further followed.

### 3.1. Farms and Animals

Median age (4.6/4.4 years), median lactation number (3/3), median milk yield per 305 d (8994/8198 kg), and breeds (Holstein Friesian, Red Holstein, and Swiss Fleckvieh) were not statistically different between the MOD and ROUT groups. Neither median BCS after calving (3/3) nor BCS-drop (0/0) from calving to conception were different between groups.

### 3.2. COF

The incidence of COFs was 25.7% in the study presented here. No significant differences were found between groups (MOD/ROUT) for cyst location (left ovary (29/28), right ovary (30/48), or both ovaries (5/4)) or type of cyst (luteinized (27/21), follicular (36/36), or both (1/0)). For the ROUT group, data concerning cyst location and cyst type in 2/25 animals was not available. The median diameter of cysts (33.5/30 mm) was larger in the MOD group (*p* = 0.0084).

### 3.3. Outcome

Fourteen days after treatment, cyst recovery was confirmed in 88.33% (MOD) versus 88.61% (ROUT) of cases. The median calving-to-conception interval was 95 d (MOD) versus 113 d (ROUT), and if cut at 200 d p.p., 94 d versus 111.5 d, but this was not statistically different (*p* = 0.1198/*p* = 0.1648). The number of AIs until pregnancy cut at 200 d p.p. ranged from one to four in both groups with no statistical difference between groups (*p* = 0.53). The median number of days from treatment to pregnancy was 42 in both groups (*p* = 0.67).

**Table 1:** Median values of variables for cows in both groups (MOD and ROUT), including minimum and maximum values.

Variables	MOD	ROUT	*p*-Value
Number of cows	64	81	
Age of cows (years)	4.6 (2.0/9.6)	4.4 (1.3/12.1)	0.6765
Lactation number	3 (1/8)	3 (1/9)	0.6972
Milk yield (305 d)	8994 (5183/13,573)	8198 (4996/12,344)	0.1775
Breed			0.2525
- Holstein Friesian	27	36	
- Red Holstein	25	25	
- Swiss Fleckvieh	12	18	
- Others	0	3	
Number of AIs	1.5 (1/6)	1 (1/7)	0.4973
BCS at parturition	3 (2.75/3.5)	3 (2.25/3.5)	0.4411
BCS loss after parturition	0 (−0.25/0.5)	0.25 (0/0.75)	0.4418
Cyst diameter in mm	33.5 (20/56)	30 (20/50)	0.0084
Cyst location			0.2752
- Left	29	28	
- Right	30	48	
- Both ovaries	5	4	
Cyst type			0.6393
- Follicular	36	36	
- Luteinized	27	21	
- Both	1	0	
Cyst recovery	88.33%	88.61%	1.0000
Calving to conception in days	95 (61/252)	113 (49/255)	0.1198
Calving to conception cut at 200 d p.p.	94 (61/152)	111.5 (49/200)	0.1648
Treatment to conception cut at 200 p.p. in days	41 (11/185)	42 (1/179)	0.8700

The therapy for the MOD group consisted of buserelin and cloprostenol on day 0 followed by cloprostenol on day 14 and buserelin on day 16. The therapy in the ROUT group consisted of other therapies (e.g., GnRH alone, hCG alone, or combined protocols with an intravaginal progesterone device (EAZI-BREED CIDR 1380^®^, Zoetis Schweiz GmbH, Delémont, Switzerland)). That group of animals (n = 81) served as the routinely treated group (ROUT).

### 3.4. Results for the Outcome Cysts (COFs)

The logistic regression for COFs included the variables group, the cyst type, breed, the number of AIs, calving to conception cut at 200 d p.p., the cyst size, and therapy. The backward (and also forward) parameter selection of the logistic regression yielded only the cyst size as a significant impact factor (*p* = 0.0019). The odds ratio for this factor was 0.900 (95% c.i. (0.842, 0.962)), meaning that the larger the cyst size, the smaller the probability of not observing a cyst 14 d after treatment, with ROC = 0.714 based on 122 data.

### 3.5. Laboratory Variables (Part B of the Study)

Only one cow in the MOD group showed clinical ketosis; n = 29 cows in the MOD group had subclinical ketosis. BHB was not routinely measured in the ROUT group.

### 3.6. Progesterone Values

Progesterone values when starting cyst therapy were only analyzed in the MOD group. There were 34 cows with a progesterone value ≤ 1 ng/mL when starting the protocol and 26 > 1 ng/mL (four values missing). Only in 37 cases did the progesterone value correspond to the cyst definition by ultrasound (24 times wrong; four values missing).

### 3.7. Trace Elements

Overall, most of the trace element values were in the normal or lower normal range (Table 2). For iodine, all values measured were in the normal range; for selenium, there was one value in the lower normal range, and all others were in the normal range; for zinc, 7 cows had a deficiency, 19 cows were in the lower normal range, and the rest were normal; for copper, 13 cows were in the lower normal range, and the rest were normal; for manganese, 14 cows were in the lower normal range, and the rest were normal. Trace element levels for three cows are missing. Chi-square tests concerning outcomes (recovery 14 d after treatment) and trace element status were not significantly different between groups.

Thresholds for trace elements were the following: iodine in umol/l (>48, normal; 32–48, lower normal range; ≤31, deficiency); selenium in umol/L (>49, normal; 30–49, lower normal range; ≤29, deficiency); zinc in umol/L (>850, normal; 654–850, lower normal range; ≤653, deficiency); copper in umol/L (>766, normal; 391–766, lower normal range; ≤390, deficiency); manganese in nmol/L (>1.7, normal; 1.3–1.7, lower normal range; ≤1.2, deficiency) [39].

## 4. Discussion

The incidence of COFs in the present study was 25.7%, which is higher compared to values found in many other studies (cumulative incidence rate, 2.7% [7]; overall incidence, 7.7% [9] or 15.15% [27]). A possible explanation for the high number of ovarian cysts recorded might be that the animals in this study were closely monitored as part of the routine Herd Health Service starting 21 d p.p.

The recovery rate was nearly identical in both groups (88.33% in the MOD group vs. 88.61% in the ROUT group). This cure rate is high compared to other studies investigating therapy for ovarian cysts (cure rates reaching from 23.1 to 77.5%) [27,31,40]. Gundling et al. (2015) analyzed the efficacy of the modified ovsynch protocol (the same as used in the MOD group) versus the conventional ovsynch protocol and found a significantly better cure rate in the group of cows treated with the modified ovsynch protocol (66.2% and 23.1%) [31]. This is consistent with our own results, showing that the modified ovsynch protocol is an effective way to treat ovarian cysts in dairy cows. The conventional ovsynch protocol, although widely used for COF treatment, leads to insufficient treatment success rates, ranging from 17 to 23% [25,31,41,42]. Lopez-Gatius and Lopez-Bejar (2002) [32] compared the treatment success of the administration of GnRH alone compared to GnRH + PGF_2α_ with another injection of PGF_2α_ administered 14 d later in both groups. The COF persistence rate was lower in the GnRH/PGF_2α_ group than in the group treated with GnRH alone (16% vs. 45%) [32]. As a possible explanation, the authors postulate that GnRH and PGF_2α_ have a synergistic effect on the luteinization of follicular cysts [32].

Cyst types are normally diagnosed using ultrasound examination, but only after a comparison of their respective progesterone values is an accurate differentiation between luteal and follicular cysts possible [18]. Practitioners rarely measure plasma progesterone levels under farm conditions due to time pressure and economic reasons [41]. In the study presented here, only 61% of the cysts diagnosed were correctly differentiated by ultrasound (performed by veterinarians working daily in the fertility service) after comparing their progesterone values. Therefore, the diagnosis of follicular and luteal cysts based on rectal exanimation is insufficient, even when ultrasound is used and veterinarians are experienced. Other studies have found a positive predictive value for follicular cyst diagnoses by ultrasound of 60 to 78%, while it is 50 to 90% for luteal cysts [43]. Therefore, efficient protocols to treat cysts without differentiation are needed and valuable, as using PGF_2α_ for the treatment of follicular cysts is ineffective, and the same is true for the treatment of luteal cysts with GnRH [40]. Therefore, a modified ovsynch protocol, as used by Gundling et al. (2015), was used on the herds visited by the Herd Health Service [31].

Cyst size negatively influenced cyst recovery. This fact was already described by Drews (2006), who also showed that first insemination success, pregnancy rate, and total recovery rate were impaired in cows suffering from larger cysts (≥4.0 cm) compared to those with smaller cysts [43].

Regarding trace elements, selenium in particular has been discussed as affecting the incidence of COFs. Previous studies have claimed that the supplementation of selenium may reduce the incidence of COFs and that high blood selenium concentrations are associated with the reduced incidence of COFs [44,45]. Therefore, we assumed that we would find low blood selenium concentrations in cows with COFs. On the other hand, Mohammed et al. (1991) found that cows with a blood selenium level > 169 ng/mL had a risk of developing COFs twice as high as animals with lower blood selenium levels [46]. This statement can neither be confirmed nor refuted based on our results because there was no cow with a blood selenium level as high as 169 ng/mL or above. Contrary to our premise, only one cow had a blood selenium concentration in the lower normal range; all other cows were within physiological limits. A possible explanation might be that all farms included in the study received routine management analysis and advice from the Herd Health Service for several years, meaning that the feeding and supplementation of trace elements are supervised and, if necessary, improved regularly. Because all cows treated with the modified ovsynch protocol had a blood selenium level within the normal range, the positive effect of an adequate selenium supply on the development of COFs seems to be rather low. There are no specific studies that have investigated the effects of the other trace elements on the development of COFs. Only seven cows in our study were deficient in blood zinc levels. All the other trace elements were within the normal range; therefore, a further statement on the influence of trace elements on COFs cannot be made based on our data. Further studies should include a larger population of cows in the herds we visited.

The body condition score did not differ between groups and did not seem to have an impact on treatment success. As high milk yield is postulated to be a risk factor for the development of COFs, we assumed that a higher BCS loss p.p. may be linked to high milk yield, and therefore, cows with a higher BCS loss would be more likely to develop COFs [23]. In accordance with our results, Lòpez-Gatius et al. did not see an influence of BCS at parturition or BCS loss after parturition on the development of COF; however, they found that cows gaining weight before calving were at a higher risk of COFs [23]. Because there were no data concerning BCS prepartum in this study, it is not possible to provide an answer as to whether an increase in BCS before calving raises the risk for COFs.

Overall, there were fewer animals included in the MOD group than those calculated in advance based on the number of cows with ovarian cysts from previous years. A potential reason leading to this circumstance is that the study was integrated into routine work from the Herd Health Service on the farms. This might have led to the fact that fewer animals were treated according to the modified ovsynch protocol, as some farmers left COFs untreated or opted for the treatment option that was successfully used in the previous years on their farms.

## 5. Conclusions

In conclusion, the modified ovsynch protocol is a useful, practical option for treating ovarian cysts with the advantage of not needing to differentiate between the two cyst types. It can be applied under field conditions and allows for the possibility of a timed AI.

## Figures and Tables

**Table 2:** Results of the analysis of the trace elements.

Trace Element	Normal Range	Lower Normal Range	Deficiency
Iodine	61	0	0
Selenium	60	1	0
Zinc	35	19	7
Copper	48	13	0
Manganese	47	14	0

## Data Availability

The datasets generated and/or analyzed during the current study are not publicly available due to the individual privacy of cattle owners but are available from the corresponding author upon reasonable request.
